# Effect of per Capita Income on the Relationship between Periodontal Disease during Pregnancy and the Risk of Preterm Birth and Low Birth Weight Newborn. Systematic Review and Meta-Analysis

**DOI:** 10.3390/ijerph17218015

**Published:** 2020-10-30

**Authors:** Carmen Alba Moliner-Sánchez, José Enrique Iranzo-Cortés, José Manuel Almerich-Silla, Carlos Bellot-Arcís, José Carmelo Ortolá-Siscar, José María Montiel-Company, Teresa Almerich-Torres

**Affiliations:** Stomatology Department, University of Valencia, 46010 València, Spain; mocaral@alumni.uv.es (C.A.M.-S.); jose.m.almerich@uv.es (J.M.A.-S.); carlos.bellot@uv.es (C.B.-A.); jose.c.ortola@uv.es (J.C.O.-S.); jose.maria.montiel@uv.es (J.M.M.-C.); teresa.almerich@uv.es (T.A.-T.)

**Keywords:** periodontal disease, premature birth, low birth weight, risk factor, income level, meta-analysis

## Abstract

This work analyzed the available evidence in the scientific literature about the risk of preterm birth and/or giving birth to low birth weight newborns in pregnant women with periodontal disease. A systematic search was carried out in three databases for observational cohort studies that related periodontal disease in pregnant women with the risk of preterm delivery and/or low birth weight, and that gave their results in relative risk (RR) values. Eleven articles were found, meeting the inclusion criteria. Statistically significant values were obtained regarding the risk of preterm birth in pregnant women with periodontitis (RR = 1.67 (1.17–2.38), 95% confidence interval (CI)), and low birth weight (RR = 2.53 (1.61–3.98) 95% CI). When a meta-regression was carried out to relate these results to the income level of each country, statistically significant results were also obtained; on the one hand, for preterm birth, a RR = 1.8 (1.43–2.27) 95% CI was obtained and, on the other hand, for low birth weight, RR = 2.9 (1.98–4.26) 95% CI. A statistically significant association of periodontitis, and the two childbirth complications studied was found, when studying the association between these results and the country’s per capita income level. However, more studies and clinical trials are needed in this regard to confirm the conclusions obtained.

## 1. Introduction

Periodontal diseases comprise a wide range of inflammatory disorders that affect the supporting structures of the tooth and can lead to both tooth loss and contribute to systemic inflammation; the disease begins and spreads due to the interaction between a dysbiosis of the oral microbiota and a vulnerable host immune system [1,2].

The initial stage of this disease is called “gingivitis”, which corresponds to a localized inflammation of the gum, with redness and/or bleeding, due, in most cases, to the deposit of bacterial plaque due to poor oral hygiene. If gingivitis is left untreated, it may progress to chronic periodontitis, characterized by a loss of the gum, bone, and periodontal ligament, resulting in what are known as periodontal pockets. The severity of periodontal disease depends on host and environmental risk factors, whether modifiable (tobacco and alcohol consumption, for example) or non-modifiable (genetic susceptibility) [1,3].

Symptoms of periodontitis include redness, change in texture and inflammation of the marginal gingiva, bleeding when probing the periodontal pocket, destruction of tooth support structures, recession of the marginal gingiva, increased morbidity and dental mobility and ultimately, tooth loss [1]. Oral diseases such as gingivitis and periodontitis are the most prevalent microbial diseases [1]. Furthermore, its relationship with numerous chronic systemic diseases makes periodontal disease an important public health problem [2,4].

At the European level, approximately 50% of the adult population suffers from some form of periodontal disease and between 10–15% suffer from severe periodontitis [5]. Periodontal diseases have a significant impact on the quality of life; individuals with this disease tend to have a negative perception of their oral health status, as well as a worse quality of life when compared to healthy individuals [2,3,6].

In recent decades, there has been a growing interest in the study of the oral microbiota and its important role in systemic health [7]. Gram-negative anaerobic bacteria are the most periodontopathogenic. Among them, the Socransky red complex (*Porphyromonas gingivalis, Tannerella forsythia* and *Treponema denticola*) and *Fusobacterium nucleatum*, while *Prevotella intermedia* and *Actinobacillus actinomycetemcomitans* stand out. The latter is more associated with aggressive periodontitis, while the former with chronic periodontitis [8,9,10].

The presence of an oral infection such as periodontal disease can be a cause or exacerbation of various systemic conditions. It is unknown whether the presence of periodontal disease together with any systemic disease is merely accidental, occurring simultaneously due to sharing similar risk factors [2,7] or if there is a causal association. If this were the case, periodontal disease could result in an increased risk of systemic diseases, aggravating or even initiating them [7].

Over the years, more evidence has emerged that local infectious diseases such as periodontitis can influence a wide variety of systemic diseases [4,11]. Dental plaque bacteria enter the bloodstream through discontinuities in oral tissues (ulcerated sulcular epiletium, infected root canals) and travel via the bloodstream to cause infection in areas far from the oral cavity. It is also possible that periodontal bacteria stimulate the release of pro-inflammatory cytokines in distant places (arteries, pancreas, liver, etc.), which may initiate or intensify a systemic pathological process (atherosclerosis, diabetes, cirrhosis, etc.), or cause inflammation or local infection in these places (pneumonia, gastric ulcers) [4].

Chronic oral infections, particularly periodontitis, could be the underlying cause of various fatal systemic diseases, such as endocarditis. Numerous studies show that heart conditions such as coronary heart disease, acute myocardial infarction, coronary infection, cardiomyopathies, peripheral vascular disease, and atherosclerosis are related to chronic processes of infection and inflammation [1,2,12,13]. Furthermore, an association has been observed between periodontitis and endothelial dysfunction, which favors vascular involvement [11,14]. It has been observed that individuals with severe chronic periodontal disease have a significantly increased risk of developing coronary heart diseases, including atherosclerosis and myocardial infarction [11,15]. Likewise, other research studies reveal the important role of periodontitis in regulating many systemic diseases, such as diabetes, chronic respiratory diseases, rheumatoid arthritis, osteoporosis, and even dementia [1,2,3,4,8,11,12,14,16].

Two pathogenic mechanisms are proposed that may explain the potential effect of periodontitis on pregnancy outcomes: On the one hand, the periodontopathogenic bacteria that inhabit the gingival biofilm, due to a translocation phenomenon, can directly affect the fetus due to bacteremia [9,10]. On the other hand, the inflammation mediators secreted in the subgingival inflammation zone (IL-1, IL-6, IL-8, TNF-alpha, prostaglandin E2) can affect the placental-fetal unit and produce an inflammatory response [8]. However, there is controversy regarding the efficacy of periodontal treatment during the pregnancy stage, since it is influenced by numerous factors such as the severity of the disease, the composition of the individual’s microbiota, the treatment strategy, and its application during the pregnancy stage [10].

During pregnancy, hormonal changes increase vascular permeability in the gingival tissues, which facilitates the diffusion of pathogenic microorganisms and their products to the circulation and to the placenta [3,9]. In addition, along with these changes in gingival vascularization, a change in the bacterial flora occurs, increasing the number of anaerobic bacteria. In the placenta, these microorganisms and their products can stimulate immune and inflammatory responses, leading to high levels of pro-inflammatory cytokine secretion in fetal tissues. This can cause premature rupture of the fetal membranes and lead to uterine contractions which, in turn, can lead to abortion or premature delivery [2,3,17].

Every year, around 15 million premature babies are born in the world. Preterm delivery is considered as delivery occurring before 37 weeks of gestation. These children, moreover, tend to be born with low weight, considered to be less than 2500 g [10]. Low birth weight preterm infants are a major cause of infant morbidity and mortality. Risk factors contributing to these adverse pregnancy outcomes include low socioeconomic status, race, multiple births, maternal age, history of preterm birth, drug and alcohol abuse, and systemic infection of the mother [4,10,18]. More than 60% of global preterm births occur in Africa and South Asia, but preterm birth is truly a global problem [10].

Not only can pregnancy interfere with the periodontal disease, but it can also alter its progression. The physiological changes induced during pregnancy can alter the inflammatory response, amplifying gingival inflammation. Gestational gingivitis affects 36–100% of pregnant women. Clinical parameters such as bleeding on probing and pocket depth can be increased without the need for an increase in plaque index and then decreased after delivery. The mechanisms by which the severity of periodontitis increases during pregnancy have been related to increased vascular permeability, depression of the immune system, and changes in the composition of the supra and subgingival microbiota [10].

Case-control studies show that women with periodontitis are more prone to preterm delivery and low birth weight [19,20,21,22,23]. Despite this, it is still not clear whether adverse pregnancy outcomes are causally related to periodontitis or are caused by another maternal or social factor [17]. Despite these epidemiological associations, the exact mechanisms by which these relationships occur remain unknown [15] but there are some factors that could explain that relationship. Chronic periodontitis is responsible for the production of C-reactive protein (CRP), interleukin-1b, interleukin 6, TNF-alpha (Tumor Necrosis Factor), as well as the dissemination of these inflammatory mediators throughout the body. These mediators have the potential to threaten the placental-fetal unit and increase the risk of complications in pregnancy [2,12,14,24].

The objective of this work will be to analyze all the evidence available in the scientific literature on the risk of preterm birth and/or giving birth to low birth weight newborns in pregnant women with periodontal disease.

## 2. Materials and Methods

### 2.1. Protocol and Registration

In order to carry out a standardized review and meta-analysis, as well as to minimize the appearance of bias in the review process as much as possible, the PRISMA (Preferred Reporting Items for Systematic Reviews and Meta-Analyses) recommendation of 2009 has been followed as the review protocol [25]. This study has been registered in PROSPERO, under number CRD42019120045.

### 2.2. Eligibility Criteria

Cohort studies that study the relationship between periodontal disease in pregnancy and preterm delivery and/or low birth weight were selected. To facilitate the study of this association, only those studies that included the relative risk values and their confidence interval have been considered. No study has been ruled out due to the language of publication; all have been published in English or contained a translation in this language.

The purpose of most of the studies was to determine the association between pregnant women with periodontal disease and the risk of premature delivery and/or low birth weight [26,27,28,29,30,31], or to analyze more generally the possible negative effects that a pregnancy with periodontitis in the woman may have on fetal development [32,33,34,35,36].

### 2.3. Information Sources and Search Strategy

The PICO (Population/Income/Comparison/Outcome) question was set up as follows: Whether there was a higher risk of preterm delivery and/or low birth weight in the population of pregnant women with periodontal disease compared to the population of healthy pregnant women.

This was followed by a systematic search for studies in three databases (PubMed, Embase, and Scopus), together with a manual search of the bibliographic references of the studies selected in the databases. It was possible to obtain the full texts of all the articles without needing to contact their authors.

The search strategy used in PubMed was: ((preterm birth) OR low birth weight) AND periodontal disease, obtaining 667 articles.

The search strategy used in Scopus was: (TITLE-ABS-KEY (preterm AND birth) OR TITLE-ABS-KEY (low AND birth AND weight) AND TITLE-ABS-KEY (periodontal AND disease)) obtaining 683 articles.

The search strategy used in Embase was: (‘premature labor’ OR ‘low birth weight’) AND ‘periodontal disease’, revealing 580 articles.

The date of the last search was 6 May 2020.

The keywords were “preterm birth” or “premature labor”, “low birth weight” and “periodontal disease”.

### 2.4. Selection of Studies

The inclusion criteria consisted of cohort studies that analyzed the relative risk of preterm delivery and/or low birth weight in pregnant women with periodontal disease. Those that only describe the possible association between these variables have been accepted, as well as those in which periodontal treatment has been carried out during pregnancy to observe the influence of this on the risk variable for preterm birth and/or periodontal disease and its comparison with the cohort of pregnant women with untreated periodontitis. The women studied had to be clinically diagnosed with periodontitis during the course of their pregnancy. Exclusion criteria were not determined.

### 2.5. Data-Gathering Process

Two calibrated reviewers (C.A.M.-S. and J.E.I.-C.) performed the database search and selection of articles for inclusion in the study. In case of discrepancy, a third reviewer (J.M.M.-C.) decided to include or exclude the article in the study. A kappa value of 0.87 was obtained to determine the inter-reviewer reliability. After reading the title and abstract, a first screening was carried out, eliminating those studies that did not meet the inclusion criteria. Subsequently, a reading of the full text of the articles that had not been discarded was made, finally selecting those that met all the eligibility criteria.

### 2.6. Data Mining

Six main sets of data have been extracted from the articles, from which their study will allow us to answer our PICO question.

- Author/year.
- Country. This will make it possible to study a possible relationship between the results obtained and the level of income per capita in the region.
- Sample size. The greater the number of patients studied, the more representative the results obtained will be. A distinction has been made between the population of pregnant women with periodontitis and those without this disease.
- Population characteristics. Weeks of gestation and mean age of the patients studied.
- Definition of periodontal disease.
- Definition of premature delivery and/or low weight.
- Relative risk (RR) of preterm birth (PB) and/or low birth weight (LBW).
- Conclusions. Whether or not an association has been observed.

### 2.7. Bias Risk in Individual Studies

The Newcastle-Ottawa scale has been used to assess the quality of the cohort studies, seeking to incorporate quality assessments in the meta-analysis interpretation of the results obtained. It is made up of eight items, divided into three dimensions (selection, comparison, and results).

The selection criterion is made up of four items, so it will have a maximum score of four points. These are: The representativeness of the exposed cohort, description of the selection of the unexposed cohort, verification of the exposure, and whether the outcome of interest was not present at the beginning of the study.

The comparison criterion is made up of a single item, but the maximum score that can be obtained in it confers two points. This item assesses whether a comparison of the cohorts has been made in the study.

The exposure criterion is made up of three items: Assessment of results, sufficient follow-up for the appearance of results, and adequate cohort follow-up. Its maximum score is three points.

Ultimately, the maximum evaluation that an article can obtain will be 8 points. Depending on the total points obtained, the article will be of greater or lesser quality. Those articles whose total sum ranges from 8 and 7 will be of high quality; of medium quality are those ranging between 6 and 5, while low quality ones have a score of less than 5.

### 2.8. Synthesis of Results

The data of this study have been analyzed with the statistical program “Comprehensive Metanalysis 3.0”.

The heterogeneity of the meta-analyses was quantified using the I^2^ statistical index, Q test, and *p*-value. Statistical heterogeneity tries to quantify the variability of the result measured in the different studies with respect to the average global result and determine if the said variability is higher than what would be expected by pure chance.

The I^2^ parameter indicates the proportion of the variation between the studies with respect to the total variation; that is, the proportion of the total variation attributable to heterogeneity. It relates the variability between studies with the internal variability in the studies. When its value falls within the range between 25 and 50, it has mild heterogeneity, while between 50–75 it is considered moderate, and if it is greater than 75, it denotes high heterogeneity.

Regarding the Q test and *p*-value, it is considered that there is heterogeneity when the value of “*p*” is less than 0.1.

Finally, the studies will be combined using the random effects model, which considers that there is an intra- and inter-study variability.

### 2.9. Bias Risk between Studies

Publication bias occurs when study results are selectively published, leading to an inadequate estimate of the true effect of an intervention.

To assess the symmetry between the studies, the risk of bias was rigorously quantified in each of the meta-analyses using a “Funnel plot” or funnel diagram, “Classic Fail-Safe Number”, “Egger’s regression intercept”, and “Duval and Tweedie’s trim and fill”.

The “Funnel plot” includes the standard error on its abscissa axis and, on the ordinate axis, the parameter to be analyzed for each study (in this case, the relative risk). In this way, a funnel-shaped figure is obtained, which allows a visual assessment of the risk of bias.

The “Classic Fail-Safe Number” indicates the number of studies that would be necessary for the results obtained to cease being significant.

“Egger’s regression intercept” is a regression method on graphs. Obtains, among other data, the standard error, and its confidence interval. If the numbers that make up this interval comprise 0, it means that there is no publication bias.

The “Duval and Tweedie’s trim and fill” allows us to analyze the existence of differences between the risk estimate obtained with imputed studies and the original. Its results are obtained by means of an imputed Funnel plot.

### 2.10. Additional Analysis

As an additional method of analysis, a meta-regression was performed to assess the possible influence of the socio-economic level of the country on the results obtained; that is, if in countries with a lower socioeconomic level there is a higher incidence of premature births or low birth weight in pregnant women with periodontitis, than in countries with a higher per capita income. To carry out the study of this association, a Scatter plot was performed.

## 3. Results

### 3.1. Selection of Studies

**Figure 1:**
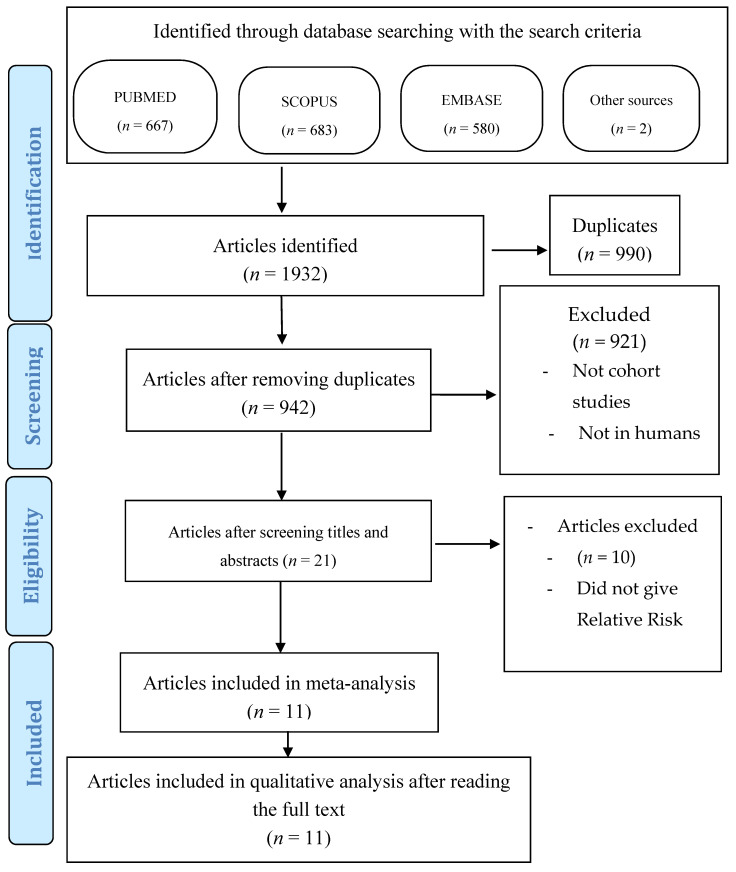
Flow diagram.

From this initial search, 1930 articles were obtained (Figure 1). Two studies were manually identified, obtained from the bibliographic review of one of the articles obtained from the database.

When comparing the articles obtained in each database, all duplicates were eliminated, leaving a total of 942 articles to review. After reading the title and “abstract” this number was reduced considerably, since only cohort studies were chosen that gave data on relative risk (inclusion criterion), reducing the total number of articles to 21.

Of these, after reading them in detail, 10 were eliminated, as they did not meet any of the inclusion criteria; three of them for not answering our PICO question, and the remaining one for giving erroneous relative risk results.

In the end, a total of 11 studies entered the meta-analysis; the full text was available for all of them.

All the articles included in the work are, as already mentioned, observational cohorts. Therefore, all of them include a control group of pregnant women without periodontitis, except for Macones et al. and Michalowicz et al. [29,36], whose control group is pregnant women with periodontitis, but not subjected to periodontal treatment during pregnancy.

The sample size of each study is considerable, ranging from 200 to 1600 pregnant women, all of whom were followed up. The age of the pregnant women included in the studies varied between 18–40 years and their onset of participation in the study ranged from week 6 to 24 of gestation.

All the examiners diagnosed periodontal disease through clinical examinations assessing attachment loss, probing depth, and number of affected teeth. In general, periodontitis was diagnosed when there was an attachment loss of ≥3 mm in ≥3 teeth.

In all cases, premature birth was established as: When the child is born before 37 weeks of pregnancy, except the one carried out by Macones, who established it at 35 weeks.

Only seven of the studies [26,28,29,30,31,34] also assessed the relative risk for low birth weight and all of them defined it as less than 2500 g.

Of the 11 articles analyzed, six concluded a statistically significant relationship between periodontal disease during pregnancy and preterm birth (López et al., Offenbacher et al., Saddki et al., Rakoto-Alson et al., Vogt et al., and Tellapragada et al.) [26,27,28,30,31,34], while the remaining five (Michalowicz et al., Srinivas et al., Pare et al., Macones et al., and Soucy-Giguère et al.) [29,32,33,35,36], which did not observe this association, were carried out in countries with higher economic incomes (United States and Canada) (Table 1).

To assess the quality of the articles included in the work, the Newcastle-Ottawa Scale for cohort studies was used (Table 2). Seven of the articles included were of very high quality (8 stars), since they meet all the requirements established for each criterion. Three were of high quality (7 stars) and the remaining one was of medium quality (6 stars).

All of them are considered to meet the four items that made up the selection criteria. Regarding the comparability criterion, only two articles did not show the comparison data between the two cohorts (Michalowicz et al. [36] and Soucy-Giguère et al. [35]).

Regarding the exposure criterion, we found more different results. In five of the articles [31,33,34,35,36] an evaluation of the results was not carried out since it was not described how the information was obtained regarding them. In all of them, there was an adequate and sufficient follow-up period for the appearance of results; this period ended at the end of the participating woman’s pregnancy.

### 3.2. Results of the Individual Studies

For the meta-analysis of periodontal disease and preterm birth, the I^2^ value was 78.2% and *p* = 0.000; in addition, the value of the Q test was 45.9. Therefore, it can be said that it has a high level of heterogeneity. In this meta-analysis, a statistically significant relative risk was obtained: 1.67 (1.17–2.838) with a 95% confidence interval, indicating a greater probability of occurrence of preterm birth in pregnant women with periodontal disease.

On the other hand, for the meta-analysis of periodontal disease and low birth weight, the results were I^2^ = 59.1% with *p* = 0.032 and Q test value = 12.2. This indicates a moderate level of heterogeneity. A statistically significant relative risk was obtained: 2.54 (1.61–3.98) with a confidence interval of 95%, indicating a higher probability of occurrence of low birth weight in pregnant women with periodontitis.

Results quite similar to those of preterm birth were observed: Studies carried out in countries with a high socioeconomic level did not establish a statistically significant relationship between periodontitis and low birth weight, while studies carried out in countries such as Chile, Malaysia, Brazil, Madagascar, and India concluded significant relationships between one variable and another.

### 3.3. Synthesis of Results

**Figure 2:**
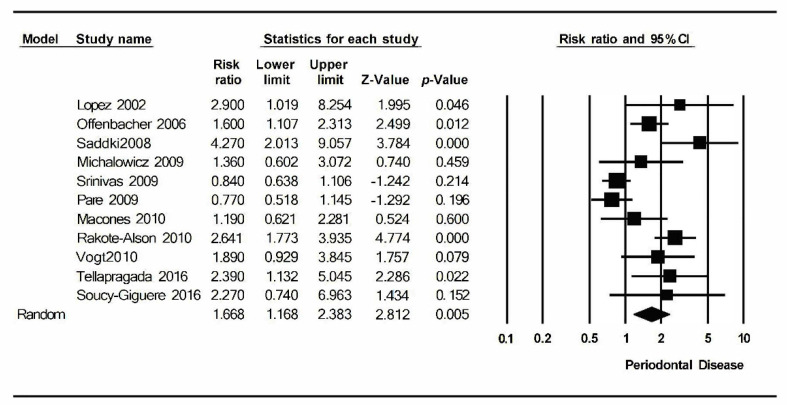
Forest plot of preterm labor.

Eleven studies have been included in the meta-analysis that relates periodontal disease in pregnant women with the risk of preterm birth. These have been combined using the random effects model, estimating a RR of 1.67 (1.67–2.38) with a 95% confidence interval (Z-value: 2.8; *p*-value: 0.005). It shows a statistically significant relationship between periodontitis and preterm birth (Figure 2).

The meta-analysis has turned out to be heterogeneous: An I^2^ value = 78.2%, Q test = 45.9 with *p*-value = 0.000; has a high level of heterogeneity.

**Figure 3:**
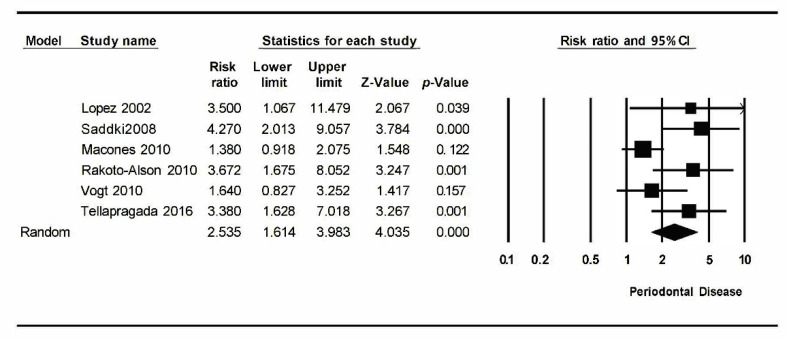
Forest plot low birth weight.

For the meta-analysis that relates periodontal disease in pregnant women with the risk of low birth weight, six studies have been analyzed. Combined using the random effects model, a RR of 2.54 (1.61–3.98) with a 95% confidence interval (Z-value: 4.03; *p*-value: 0.000) has been estimated. It shows a statistically significant relationship between periodontitis and low birth weight (Figure 3).

The meta-analysis turned out to be heterogeneous: An I^2^ value = 59.72% was obtained, Q test = 12.25 with *p*-value = 0.032. It has a moderate level of heterogeneity.

### 3.4. Bias Risk among Studies: Analysis of Publication Bias

For preterm birth, a standard error funnel plot was performed to assess whether there was symmetry between the studies. Graphically, a symmetric distribution of the studies is observed along the funnel; that is, there is no publication bias.

The Classic Fail-Safe Number was then performed for preterm labor. The result is that 67 more studies would be necessary for the labor results regarding preterm birth for it to cease to be significant, which supports the significance of the relative risk obtained in the Forest Plot of Figure 1. There is no bias of publication.

In the Egger’s regression intercept for preterm birth, the result was 2.75 (−0.29–5.08), with a *p*-value of 0.07. Since the confidence interval includes values that pass through 0 and the *p*-value is statistically significant, it was considered that there was no publication bias.

**Figure 4:**
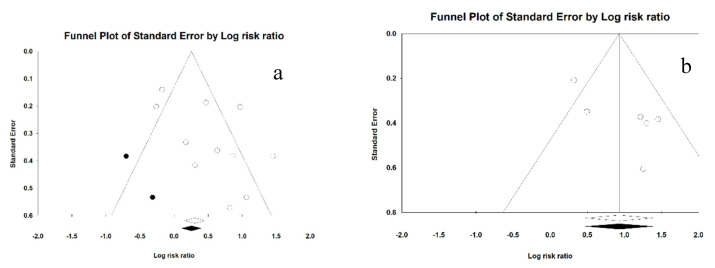
Funnel plot for preterm labor (**a**) and low birth weight (**b**).

Using the Duval and Tweedie’s trim and fill for preterm labor, we obtained the imputed Funnel plot. Obtained as adjusted RR 1.46 (1.03–2.06); no publication bias was detected (Figure 4a).

For low birth weight, a standard error Funnel plot was performed to assess the symmetry between the studies. Graphically, a symmetrical distribution of the studies was observed along the funnel; that is, there is no publication bias.

Regarding the Classic Fail-Safe Number for low birth weight, it was established that 56 articles were necessary for our results so that this variable ceased to be significant. This finding supports our assertion that there is no publication bias.

In the Egger’s regression intercept for low passage at birth, the result was 3.63 (−0.05–7.31), with a *p*-value of 0.052. The confidence interval and the *p*-value comprise more adjusted values than those obtained for preterm delivery; there is greater exposure to bias, but it can be concluded that there is no bias.

Using the Duval and Tweedie’s trim and fill for low birth weight, we obtain the Funnel plot. It is obtained as adjusted RR 2.54 (1.61–3.98); no study could be identified, so there is no publication bias (Figure 4b).

### 3.5. Additional Analysis: Meta-Regression

The relationship between the risk of preterm birth in pregnant women with periodontitis and the income level of the country of each article in the study was analyzed using a meta-regression. The 2018 “World Bank list of economies” was used to classify the countries in which the different studies were carried out according to their per capita income.

Countries were categorized according to their income. Thus, a value of 2 was given to the countries with a low-to-middle socioeconomic level (per capita income: $996–3895; India), 3 to those with a medium-to-high level (per capita income: $3896–12,055; Malaysia, Brazil, and Chile), and 4 to high (per capita income ≥ $12,056; United States and Canada), taking as reference the value of 1 = very low socioeconomic level (income ≤ $995; Madagascar).

The R^2^ indicates the predictive capacity of the linear meta-regression model that has been generated; in this case it was 84%. The significant result occurs in group 4 of per capita income, with a coefficient of -0.89 and a value of *p* = 0.002. This means that the RR of preterm birth decreases 0.89 times in high-income countries (Table 3).

**Figure 5:**
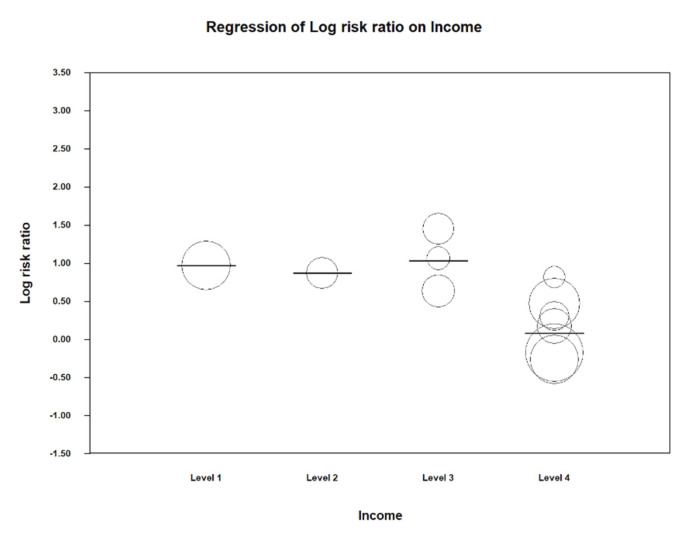
Scatter plot for preterm labor and socio-economic level of the country.

In the Scatter plot of preterm birth and socioeconomic level, 1 is taken as a reference and corresponds to a low socioeconomic level. It is observed how at level 4 the risk of preterm birth is greatly reduced; it follows that in high-income countries the relative risk of this event is significantly lower (Figure 5).

The following table shows the estimated data on the relative risk of preterm birth for each economic subgroup. It is observed how this value decreases as the per capita income of the country increases; however, in level 4, with a confidence interval of 0.86–1.69 and a value of *p* = 0.25, a statistically significant result is no longer obtained. This means that there is no risk of preterm birth in high-income countries (in this case, those included in this group were the USA and Canada) (Table 4).

Likewise, another meta-regression analysis was carried out that related the socioeconomic level of the countries included in the study with the relative risk of LBW. The countries were categorized according to their income following the same previous model: A value of 2 for the countries with a low-to-middle socioeconomic level (India), 3 for the medium-to-high (Malaysia, Brazil, and Chile), and 4 for the high (USA and Canada), taking the value of 1 as a reference = lowest level (Madagascar).

The R^2^ indicates the predictive capacity of the linear meta-regression model that has been generated; in this case it was 100%. The significant result occurs in group 4 of income per capita, with a coefficient of −0.98 and a value of *p* = 0.03. This means that the RR of preterm birth decreases 0.98 times in high-income countries (Table 5).

**Figure 6:**
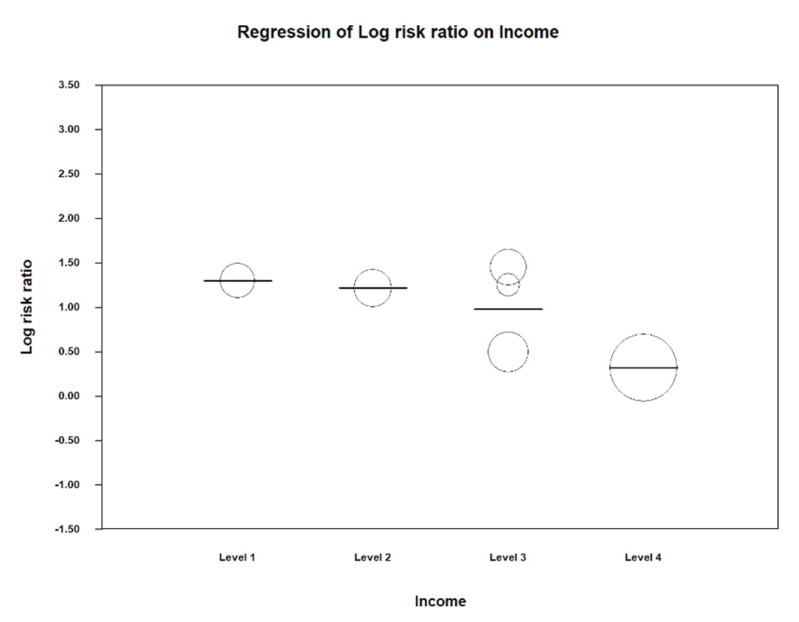
Scatter plot for low birth weight and socio-economic level.

In the Scatter plot of LBW and socioeconomic level, the value of 1 is taken as a reference, which corresponds to a low socioeconomic level. At level 4 we can observe that the risk of LBW is significantly reduced; it can be deduced that in high-income countries, the relative risk of this event is significantly lower (Figure 6).

The following table shows the estimated data on the relative risk of low birth weight for each economic subgroup. The trend is as clear as in preterm birth, because the risk decreases as the income level rises. This means that there is no risk of low birth weight in high-income countries (in this case, those included in this group were the USA) (Table 6).

## 4. Discussion

In recent years, there has been an increase in scientific evidence suggesting an association between oral health status, specifically periodontal disease, and an increased risk of systemic diseases. These diseases are multiple and varied: Atherosclerosis, hypertension, infective endocarditis and other cardiovascular diseases, erectile dysfunction, rheumatoid arthritis, chronic kidney disease, diabetes mellitus, and various adverse pregnancy outcomes, such as an increased risk of low birth weight babies, childbirth prematurity, preeclampsia, and gestational diabetes [8,37].

Regarding the reason for the relationship between periodontitis and premature delivery and/or low birth weight, it seems that the hypothesis most supported by the medical literature is that of inflammatory processes in the placental-fetal junction or elevated systemic inflammation (situations that can be caused by periodontal disease in the pregnant mother) can produce these adverse effects in childbirth [37].

Furthermore, the presence of periodontal disease in pregnancy can lead to the translocation of periodontopathogenic bacteria to the utero-placental circulation. This bacterial accumulation can provoke a response from the fetal membrane, whose functions may be interrupted and, therefore, predispose the said membrane to rupture and premature delivery [38,39].

It could be said that the relationship between periodontal disease and pregnancy is bidirectional as the latter is also strongly related to the incidence periodontal disease. The increased hormonal level of estrogens and progesterone that occurs in pregnant women causes an increase in the blood supply to the gum tissue, resulting in inflammation and bleeding of the gums. This situation of gingivitis, maintained over time, will evolve into periodontitis [40].

A premature newborn is one that is born before completing the 37th week of gestation. The difficulty of knowing the gestational age of the newborn makes it necessary to use birth weight as a reference parameter. The neonate is classified as underweight when its birth weight is less than 2500 g; very low weight when it is less than 1500 g and, extremely low weight, when it is less than 1000 g [41].

The identification of young children for their gestational age (SGA; those with low birth weight) is important because they present an increased risk of perinatal morbidity and mortality, and of cardiovascular disease in adulthood [41]. Low birth weight newborns are 40 times more likely to die than those born with normal weight, and this risk increases even more when it is associated with complications derived from preterm birth, such as chronic lung disease or respiratory stress syndrome [42].

By establishing the relationship between the parameters of weight and gestational age, we can subdivide the preterm population into high weight, adequate weight, and low weight for their gestational age, a situation that will determine the probability of postnatal morbidity [43].

Prematurity is a serious public health problem due to the great morbidity and mortality it generates, being the leading cause of mortality in children under 5 years of age, in addition to the high economic and social costs that its care causes [44]. Most of the morbidity and mortality affects “very preterm” newborns, whose gestational age is less than 32 weeks, and especially the “extreme preterm” that are those born before the 28th week of gestational age [43].

According to the World Health Organization (WHO), it is estimated that 15 million premature babies are born annually worldwide; that is, one in 10 births is premature. Of these, approximately one million die each year due to complications from this preterm delivery. Of the babies that survive, many develop some type of permanent disability, mostly related to learning, visual, or hearing problems.

The determining factors of this complication in pregnancy are multiple. In preterm birth, in addition to biological determinants, those that are the responsibility of the health of each country and the state are involved (highlighting political, environmental, social, and economic factors). As it is a public health problem, the prevention and treatment of prematurity should be an obligatory public policy [44]. The strategies used to improve prenatal, medical, dietary care, and social care are effective in controlling prematurity rates [41].

It has also been suggested that social and psychological factors affect health behavior and this in turn affects periodontal disease. These factors include oral hygiene, dental health monitoring, and smoking, and are related to both socioeconomic conditions and periodontal disease [45].

Regarding the etiopathogenesis of low weight for gestational age of the newborn child, maternal, placental, and fetal causes have been described as factors involved in this event. However, in most cases the cause is not clear. Maternal factors seem to be involved in half of the cases, highlighting in developed countries the importance of severe gestational hypertension, the history of a previous SGA child and, as a preventable and avoidable cause, maternal smoking stands out [41].

The fundamental objective that was the basis for the start of the work has been fulfilled. On the one hand, a statistically significant relationship has been established between periodontal disease in pregnancy and preterm delivery. Based on the results obtained, the fact that the pregnant mother suffers from periodontal disease increases the risk of premature birth 1.67 times; RR = 1.67 (1.17–2.38), 95% CI.

On the other hand, regarding the possibility of giving birth to a low-weight baby in women with periodontitis, this relationship was also demonstrated with a relative risk value of 2.53 (1.61–3.98), 95% CI.

Therefore, the findings of this work indicate that the presence of periodontal disease in the pregnant mother is a determining risk factor when suffering such adverse outcomes in pregnancy.

Within the classification of levels of evidence according to the Oxford Centre for Evidence-Based Medicine (CEBM), a meta-analysis of cohort studies corresponds to a high level of 2a evidence. According to the modified GRADE system, we have a grade of recommendation 1B. This indicates a strong recommendation for the results obtained and evidence of moderate quality.

The advantage of using only cohort studies in the meta-analysis is that they will allow the results obtained to be extrapolated more easily to the general population, giving greater external validity.

The fact that only published studies had been analyzed could have led to an overestimation of the risk caused by periodontal disease for the pregnant mother, but this is not the case in this study, since we have estimated this publication bias and found that it is non-existent.

We think that the work provides a good estimate of the association between periodontal disease and the two study variables, since the sources of variability in the results of the different studies have been quantified.

However, the limited number of studies in the scientific literature on this topic has determined that the number of articles analyzed has been limited, this being a possible bias for the results obtained.

This meta-analysis differs from others already carried out [46,47] by focusing only on cohort studies and, specifically, those that include relative risk values. This has allowed us to establish a relationship more easily between the study variables. Furthermore, most of the articles included in this meta-analysis are more recent than the previously conducted meta-analyses. Despite this, these meta-analyses also concluded an association or, at least, a tendency to suffer adverse effects in pregnancy when the pregnant mother suffered from periodontitis.

Heterogeneity has been controlled by combining the studies using the random effects model, which allows it to be assessed by both an intra- and inter-study. One of the main sources of heterogeneity that has been studied has been the country in which each study was carried out; specifically, its per capita income level.

Already in the meta-analysis carried out by Vergnes and Mixou in 2007 [46] the economic factor stands out as an important determinant in the results of the risk of preterm birth.

The country’s income level not only determines an increased risk of preterm or low birth weight, but also influences the survival of that child born prematurely. The WHO estimates that in low-income countries, half of preterm infants die because they have not received simple care such as breastfeeding or basic care to avoid infections and respiratory problems; in contrast, in high-income countries most of these babies survive.

In this study, we wanted to establish if there could be a relationship between the increased risk of premature birth in pregnant women with periodontal disease and the income level of each country; for this, a meta-regression was carried out. The existence of a statistically significant association was determined, with RR: 1.8 (1.43–2.27), CI 95%. The risk of suffering preterm birth increases in countries with a lower level of income per capita, such as Malaysia, India, Chile, Brazil, Madagascar or India, and disappears in those with a high level of income that, in this work, corresponded with the United States and Canada. Likewise, it has been determined that the country’s income level is also closely related to the appearance of low birth weight, with a RR of 2.9 (1.98–4.26), 95% CI. These are very similar results to those obtained in the meta-regression for preterm birth, since the risk of low birth weight ceases to exist in countries with higher per capita income. The fact that this risk disappears in developed countries could be explained by the important role that psychosocial factors play in oral health care.

However, some studies [5,47] suggest that the difference in income would not be the only cause of worsening in health markers, as in the case at hand it would be periodontal disease or premature birth. In their studies, they found that other factors, such as the population’s level of education, could be the cause of the said worsening in health markers, so income should not be taken solely as a reference.

Despite the evidence associating periodontitis with gestational complications, the effect of periodontal treatment in pregnancy is not yet supported by scientific evidence [10,29,36]. Randomized clinical trials with large sample sizes should be carried out in which periodontal treatment protocols are tested in pregnant women [10].

### Limitations of the Study

The objective of this work was to analyze the evidence available in the scientific literature on the risk of preterm birth and/or giving birth to low birth weight newborns in pregnant women with periodontal disease, and how other factors such as the per-capita income can affect it. In our approach, we limited the data mining to relative risk, leading to include 11 studies in the final synthesis. In future studies, more variables should be included to avoid this limitation.

Another limitation of our study was the per-capita income definition. Only one study was carried in a country with socio-economic level 1. Maybe including other variables and not only the relative risk could lead to include more countries of this socio-economic level. Moreover, the definition of the socio-economic level was made through the theoretical level of the country, and maybe it is not the same in all the departments of the country.

Finally, the definition of the periodontal disease in each study is different, making the data mined heterogeneous. In future studies, the definition of this disease could be an exclusion criteria, focusing in probing depth or periodontal loss.

## 5. Conclusions

After analyzing the published literature, we can conclude that the risk of suffering preterm birth is 1.67 times higher in women with periodontitis, and the risk of a newborn with low birth weight, is 1.42 times, with a 2a evidence level.

In addition, when comparing these results with the per capita income level of each country, we have observed that the risk of suffering preterm birth in pregnant women with periodontitis increases 1.8 times in countries with lower socioeconomic levels, and seems to disappear in countries with higher per capita incomes. Similarly, the same happens with regard to the risk of giving birth to low-weight babies in pregnant women with periodontal disease: This fact is 2.9 times more likely to happen in countries with a lower economic level, and disappears in those with greater income levels. These results may not be attributed only to income, but to other factors such as education levels and therefore, this relationship should be more closely examined in future research.

## Figures and Tables

**Table 1:** Main results of the studies analyzed.

Study (Country)	Sample Size Total *n* (*n* of Patients with Periodontal Disease PD)	Sample Characteristics	Definition of Periodontal Disease	Definition of Preterm Labor (PL) and/or Low Birth Weight (LBW)	Relative riskt (RR) (95% CI) of Preterm Labor (PL) and/or Low Birth Weight (LBW)	Conclusions
López et al., 2002 (Chile) [26]	*n* = 639 (233 with PD).	<21 weeks of pregnancy. Age between 18–35 years. Low socio-economic level.	≥4 teeth with ≥1 points with a probing depth ≥4 mm and an insertion lost in that point of ≥3 mm.	PL: <37 weeks. LBW: <2500 g.	PL: 2.9 (1.019–8.254). LBW: 3.5 (1.067–11.478).	PD related with PL and LBW.
Offenbacher et al., 2006 (USA) [27]	*n* = 1020 (735 with PD).	<26 weeks of pregnancy. Age >18 years.	PD moderate-severe: ≥15 points with >4 mm of probing depth.	PL: <37 weeks. Very Preterm Labor: <32 weeks.	PL: 1.6 (1.107–2.314).	PD increases risk of PL.
Saddki et al., 2008 (Malaysia) [28]	*n* = 472 (232 with PD).	Second trimester of pregnancy.	≥4 periodontal pockets of ≥4 mm. Insertion loss of ≥3 mm with bleeding in that point.	PL: <37 weeks. LBW: <2500 g.	PL: 4.270 (2.013–9.056). LBW: 4.27 (2.01–9.04).	Statistically significant relation between PD and PL and between PD and LBW.
Michalowicz et al., 2009 (USA) [36]	*n* = 796 (796 with PD). 402 treated.	13–32 weeks of pregnancy. Age between 16–44 years.	---	PL: <37 weeks.	PL: 1.36 (0.602–3.071).	Periodontal treatment not decrease the incidence of PL.
Srinivas et al., 2009 (USA) [32]	*n* = 786 (311 with PD).	6–20 weeks of pregnancy.	Insertion loss of ≥3 mm in ≥3 teeth.	<37 weeks.	PL: 0.84 (0.64–1.11).	PD is a risk factor independent of PL.
Pare et al., 2009 (USA) [33]	*n* = 1569 (1058 with PD).	6–20 weeks of pregnancy.	Insertion loss of ≥3 mm in ≥3 teeth.	<37 weeks.	PL: 0.77 (0.52–1.150).	No association between PD and PL.
Macones et al., 2010 (USA) [29]	*n* = 757 (757 with PD). 378 treated.	6–20 weeks of pregnancy.	Insertion loss of ≥3 mm in ≥3 teeth.	<35 weeks. LBW: <2500 g.	PL: 1.19 (0.62–2.28). LBW: 1.38 (0.918–2.075).	The treatment of periodontal disease not reduce the incidence of PL nor LBW.
Vogt et al., 2010 (Brazil) [34]	*n* = 327 (156 with PD).	≤32 weeks of pregnancy. Age between 18–42 years.	≥4 teeth with ≥1 point with probing depth and insertion loss of ≥4 mm.	<37 weeks. LBW: <2500 g.	PL: 1.89 (0.93–3.85). LBW: 1.64 (0.82–3.30).	PD increases the risk of PL and LBW.
Rakoto-Alson et al., 2010 (Madagascar) [30]	*n* = 204 (47 with PD).	20–34 weeks of pregnancy.	Insertion loss of ≥5 mm in ≥3 teeth.	<37 weeks. LBW: <2500 g.	PL: 2.64 (1.773–3.936). LBW: 3.672 (1.675–8.054).	Statistically significant relation between PD, PL and LBW.
Tellapragada et al., 2016 (India) [31]	*n* = 726 (90 with PD).	8–24 weeks of pregnancy. Age between 18–35 years.	Probing depth of ≥4 mm and insertion loss of ≥3 mm in 1 of the 6 index teeth.	<37 weeks. LBW: <2500 g.	PL: 2.39 (1.1–4.9). LBW: 3.38 (1.6–6.9).	PD increases the risk of PL and LBW, but more studies are necessary.
Soucy-Giguère et al., 2016 (Canada) [35]	*n* = 258 (117 with PD).	Between 15–24 weeks of pregnancy.	≥1 points with probing depth of ≥4 mm y ≥10% with bleeding.	<37 weeks.	PL: 2.27 (0.74–6.96).	No association found between PD and PL.

**Table 2:** Newcastle-Ottawa quality scale for cohort studies.

Study	Selection (Maximum 4 Stars)				Comparability (Maximum 2 Stars)	Outcomes (Maximum 3 Stars)			TOTAL
	Representativeness of Exposed Cohort	Selection of Non-Exposed Cohort	Ascertainment of Exposure	Outcome Not Present at the Start of the Study		Assessment of Outcomes	Length of Follow-Up	Adequacy of Follow-Up	
López et al., 2002 [26]	☆	☆	☆	☆	☆	☆	☆	☆	8
Offenbacher et al., 2006 [27]	☆	☆	☆	☆	☆	☆	☆	☆	8
Saddki et al., 2008 [28]	☆	☆	☆	☆	☆	☆	☆	☆	8
Michalowicz et al., 2009 [36]	☆	☆	☆	☆			☆	☆	6
Srinivas et al., 2009 [32]	☆	☆	☆	☆	☆	☆	☆	☆	8
Pare et al., 2009 [33]	☆	☆	☆	☆	☆		☆	☆	7
Macones et al., 2010 [29]	☆	☆	☆	☆	☆	☆	☆	☆	8
Vogt et al., 2010 [34]	☆	☆	☆	☆	☆	☆	☆	☆	8
Rakoto-Alson et al., 2010 [30]	☆	☆	☆	☆	☆		☆	☆	7
Tellapragada et al., 2016 [31]	☆	☆	☆	☆	☆		☆	☆	7
Soucy-Giguère et al., 2016 [35]	☆	☆	☆	☆			☆	☆	6

**Table 3:** Results of the meta-regression between preterm labor and socio-economic level of the country.

Main Results for Model 1, Random Effects (ML), Z-Distribution, Log Risk Ratio						
Co-Variable	Coefficient	Standard Error	Lower 95%	Upper 95%	Z-Value	*p*-Value
Intercept	0.97	0.27	0.44	1.50	3.6	0.0002
Per capita income level: 2	−0.10	0.50	−1.08	0.88	−0.20	0.4210
Per capita income level: 3	0.06	0.38	−0.67	0.80	0.17	0.4339
Per capita income level: 4	−0.89	0.30	−1.48	−0.30	−2.98	0.0015

**Table 4:** Estimated risk ratio of preterm labor for each economic subgroup.

Per Capita Income Group	Risk Ratio	Lower Limit	Upper Limit	Z-Value	*p*-Value
1	2.641	1.773	3.936	4.774	0
2	2.39	1.132	5.044	2.286	0.022
3	2.813	1.266	6.252	2.539	0.011
4	1.212	0.869	1.69	1.131	0.258
Total	1.804	1.432	2.274	5	0

**Table 5:** Results of the meta-regression between low birth weight and socio-economic level of the country.

Main Results for Model 1, Random Effects (ML), Z-Distribution, Log Risk Ratio						
Co-Variable	Coefficient	Standard Error	Lower 95%	Upper 95%	Z-Value	*p*-Value
Intercept	1.30	0.40	0.52	2.09	3.25	0.0012
Per capita income level: 2	−0.08	0.55	−1.15	0.99	−0.15	0.8796
Per capita income level: 3	−0.32	0.47	−1.24	0.59	−0.69	0.4886
Per capita income level: 4	−0.98	0.45	−1.86	−0.09	−2.17	0.0302

**Table 6:** Estimated risk ratio of low birth weight for each economic subgroup.

Per Capita Income Group	Risk Ratio	Lower Limit	Upper Limit	Z-Value	*p*-Value
1	3.672	1.675	8.054	3.247	0.001
2	2.327	1.146	4.724	2.337	0.019
3	4.27	2.013	9.056	3.785	0
4	1.847	0.793	4.303	1.421	0.155
Total	2.899	1.975	4.256	5.433	0
